# Gas Migration Episodes Observed During Peridotite Alteration in the Samail Ophiolite, Oman

**DOI:** 10.1029/2022GL100395

**Published:** 2022-10-31

**Authors:** John M. Aiken, Robert A. Sohn, François Renard, Juerg Matter, Peter Kelemen, Bjørn Jamtveit

**Affiliations:** ^1^ Njord Centre Departments of Physics and Geosciences University of Oslo Oslo Norway; ^2^ Department of Geology and Geophysics Woods Hole Oceanographic Institution Woods Hole MA USA; ^3^ CNRS IRD ISTerre University Grenoble Alpes Grenoble INP University Savoie Mont Blanc University Gustave Eiffel Grenoble France; ^4^ School of Ocean and Earth Science University of Southampton Southampton UK; ^5^ Lamont Doherty Earth Observatory Columbia University Palisades NY USA

**Keywords:** serpentinization, hydroacoustics, reaction driven cracking, hydrogeology

## Abstract

Serpentinization and carbonation of mantle rocks (peridotite alteration) are fundamentally important processes for a spectrum of geoscience topics, including arc volcanism, earthquake processes, chemosynthetic biological communities, and carbon sequestration. Data from a hydrophone array deployed in the Multi‐Borehole Observatory (MBO) of the Oman Drilling Project demonstrates that free gas generated by peridotite alteration and/or microbial activity migrates through the formation in discrete bursts of activity. We detected several, minutes‐long, swarms of gas discharge into Hole BA1B of the MBO over the course of a 9 month observation interval. The episodic nature of the migration events indicates that free gas accumulates in the permeable flow network, is pressurized, and discharges rapidly into the borehole when a critical pressure, likely associated with a capillary barrier at a flow constriction, is reached. Our observations reveal a dynamic mode of fluid migration during serpentinization, and highlight the important role that free gas can play in modulating pore pressure, fluid flow, and alteration kinetics during peridotite weathering.

## Key Points

- This paper presents hydrophone array data from a borehole in an actively serpentinizing terrain contain acoustic signals from bubble swarms discharging into the hole
- Free gas accumulates in the formation and migrates rapidly in discrete swarm episodes when a pressure threshold is reached
- Results reveal a gas migration mechanism associated with peridotite alteration, a process which enhances the kinetics of peridotite weathering

## 1 Introduction

The peridotites constituting the Earth's upper mantle are highly reactive when exposed to aqueous fluids under near‐surface conditions (Kelemen & Hirth, 2012). Peridotite alteration is a major transfer process for volatiles between the hydrosphere and the lithosphere (Guillot & Hattori, 2013), and in the presence of carbon‐bearing fluids it leads to carbonate precipitation (Power et al., 2013), which represents a natural mechanism for carbon capture and storage (Kelemen & Matter, 2008). Peridotite alteration is a complex process that can proceed along several different chemical reaction paths depending on the details of the fluid and rock compositions. These reaction paths have been described (e.g., Johannes, 1969; B. W. Evans, 1977; Frost, 1985), but the physical processes associated with peridotite alteration, including how fluids migrate in the rock, how fluids penetrate into fresh rock and generate complex vein networks (Aupart et al., 2021; Iyer et al., 2008), and how these processes are affected by variations in reaction chemistry, are poorly understood.

Theoretical (Kelemen & Hirth, 2012), laboratory (Xing et al., 2018; Zhu et al., 2016), and numerical simulations (Malvoisin et al., 2020; O. Evans et al., 2020) generally assume that gases generated by peridotite alteration (H_2_ and CH_4_) are dissolved into the reaction product fluids, and that the fluids flowing through the formation are single‐phase liquids. H_2_ and CH_4_ gases can be observed bubbling up in alkaline springs in some peridotite terrains (Kelemen et al., 2021; Leong et al., 2021), but it has been hypothesized that this represents the near‐surface exsolution of dissolved gases produced at higher pressures deeper in the subsurface (Sleep et al., 2004). The fate of the gas produced by peridotite alteration, and how it is partitioned between dissolved and free gas phases, has important consequences for fluid flow. Two‐phase flows behave very differently than single phase flows (Brennen, 2005), and the competition between gas and liquid for pore occupancy can create pressure cycles that generate unsteady, episodic flow (Persoff & Pruess, 1995).

**Figure 1:**
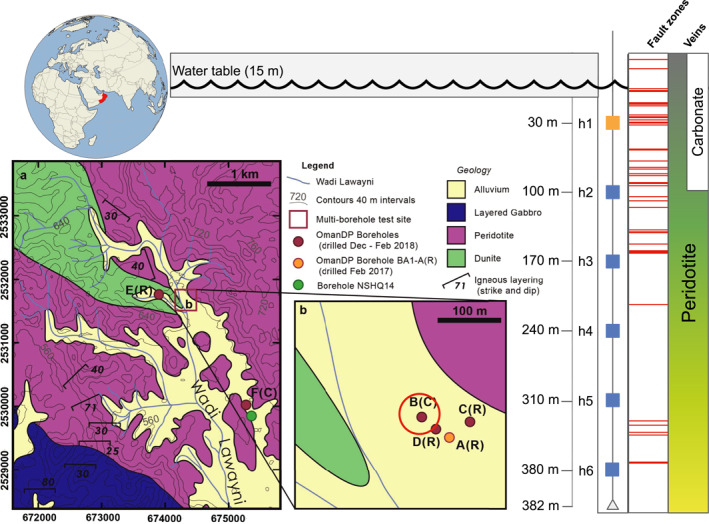
The Multi‐Borehole Observatory (MBO) site of the Oman Drilling Program. The site BA1 is located in Wadi Lawayni, Oman (22.8813°N, 58.7006°E). Four boreholes were drilled (A, B, C, D) by a combination of rotary drilling (R) and diamond coring (C). A six‐element hydrophone array (h1 ‐ h6) with 70 m inter‐element vertical spacing, sampled at 1 kHz, was deployed in Hole BA1B for 9 months. The entire 400 m depth sequence of Hole BA1B is composed of variably altered peridotite. The degree of alteration, colored from green to yellow, decreases with depth in the hole, and carbonation is restricted to the upper ∼100 m. Dense sets of fault/fracture zones (red lines) intersect the upper part of the hole. The acoustic signal from all of the bubbles detected in this study arrives at the topmost hydrophone (h1, colored orange) first.

Most peridotite terrains are located in the deep sea, making them difficult to study. However, the Oman Drilling Project (OmanDP) (Kelemen et al., 2020) has provided a unique opportunity to advance our understanding of peridotite alteration by establishing a Multi‐Borehole Observatory (MBO) in an actively serpentinizing terrain of the Samail ophiolite (Kelemen et al., 2021). Four, 400 m deep boreholes were drilled within a ∼100 × 100 m region (Figure 1), providing an opportunity to study the geological and alteration history of the uplifted mantle rocks, and to monitor presently occurring alteration processes. Here, we present hydrophone array data from one of the boreholes to show that free gas migrates through the formation in discrete swarms of activity. Gas produced by alteration and/or microbial activity accumulates in the permeable flow network, is pressurized, and discharges intermittently into the borehole when a critical pressure, likely associated with a capillary barrier at a flow constriction, is reached. Our observations highlight the important role that free gas can play in modulating fluid flow within the alteration vein network, and reveal a previously unknown episodic gas migration mechanism.

## 2 Data, Methods, and Results

**Figure 2:**
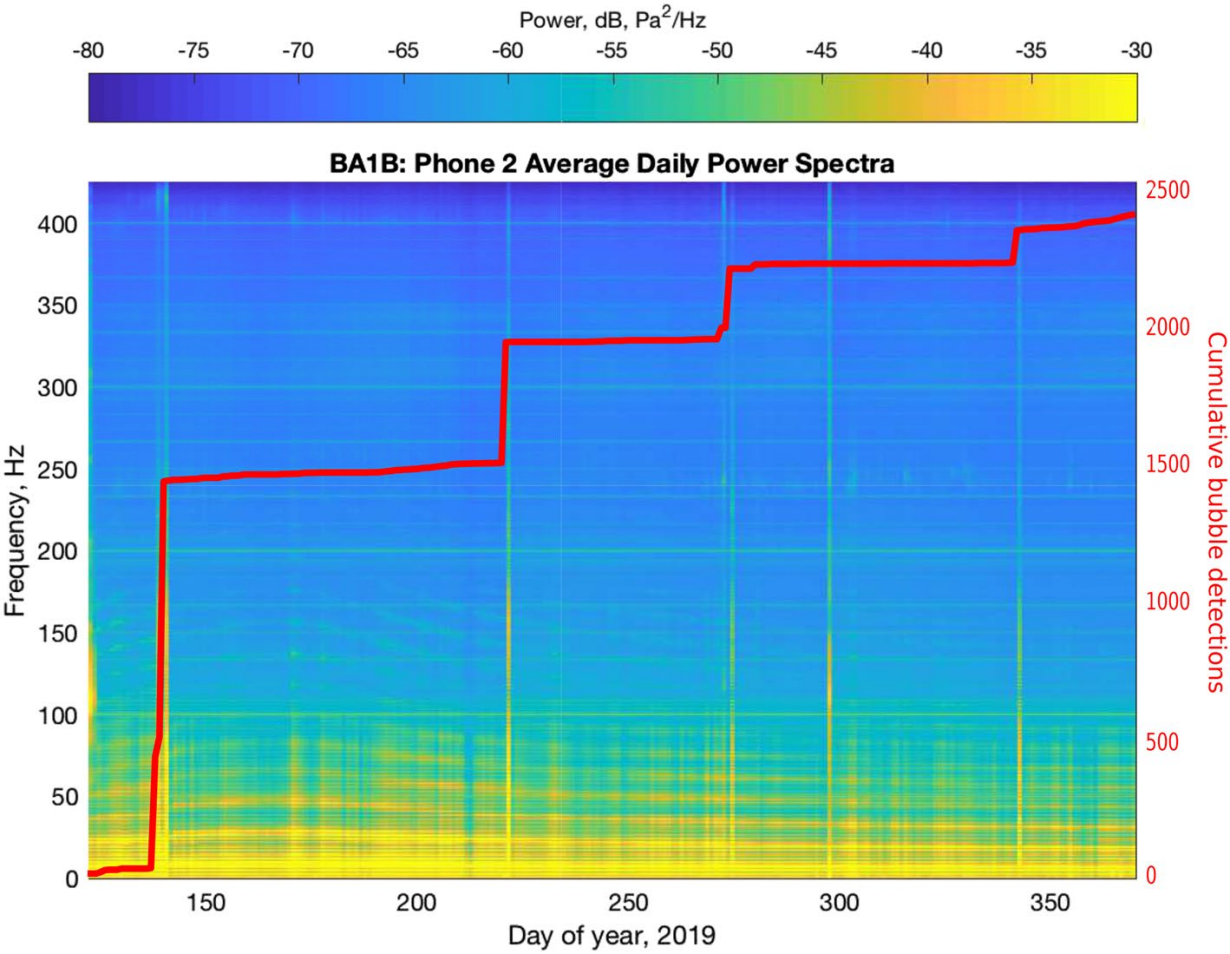
Spectrogram of time‐series data from hydrophone h2. Bubble swarms appear on this plot as vertical stripes due to their high‐frequency content. The stripe on day 297 is due to human activity at the site rather than bubbles. The bright yellow horizontal bands at frequencies ≤50 Hz are due to weakly damped water column resonant modes inside the borehole, while the fainter resonances at frequencies between ∼50–200 Hz are modes of the air‐filled portion of the hole. All of these borehole resonant modes change frequency as the water level changes. The faint, horizontal stripes at evenly spaced, constant, frequencies are due to electrical noise. The red curve shows the cumulative count of bubble detections.

We deployed an array of six, High Tech HTI‐96‐MIN hydrophones with 70 m inter‐element spacing in Hole BA1B from May 2019 to February 2020. The data were sampled at 1 kHz and recorded with a Quanterra Q330S+ data logger after applying a low‐pass, anti‐aliasing, filter with a corner frequency of 450 Hz, which sets the upper frequency resolution limit. Raw hydrophone data (digital counts) were converted to physical units (Pa), and the polarity for phone h4 was corrected (raw polarity was reversed). Power spectra for each hydrophone were estimated using multi‐taper techniques (time‐bandwidth product = 2) on 15‐min segments, and then averaging these to obtain daily power spectra estimates for the nine‐month deployment interval (Figure 2).

**Figure 3:**
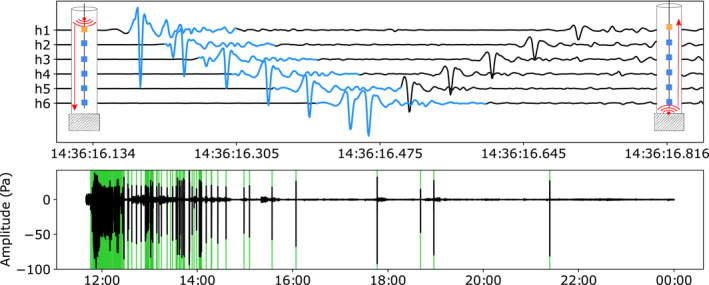
Bubble signals. Top: An example of a single bubble signal, high‐pass filtered at 40 Hz, observed on all hydrophones. The pressure signal propagates down the borehole as a tube wave, reflects off the bottom, and propagates back up again (inset schematic). The blue waveform indicates the template used to detect bubbles in the hydrophone data. Bottom: Raw data from hydrophone h1 for the 18 May 2019 bubble swarm episode. The green bars indicate bubble detections using the matched filter technique (Gibbons & Ringdal, 2006).

The spectral estimates reveal that the data records are punctuated by four, hours‐long, swarms (Figure 2) of short (∼0.2 s), impulsive signals. Each swarm contains a few smaller minutes long sub‐swarms. The signals, which are sourced near the top of the hole, propagate down and back up the borehole as Biot (tube) waves (Biot, 1952) (Figure 3). The acoustic signals are typical of gas bubbles that nucleate in, or discharge into, a liquid (Jablonská et al., 2017; Vazquez et al., 2015), with an initial, broadband impulse, followed by resonant oscillations of the newly formed bubble. The waveforms of the acoustic signals suggest that the data were not sampled fast enough to accurately capture the impulsive onset, but the sampling rate was adequate for signal identification.

All the bubble signals have similar waveforms, and we used a matched filter template detection method to generate a catalog of bubble events for the 9‐month deployment interval (Gibbons & Ringdal, 2006). We normalized the data to zero‐mean, unit‐variance, and high‐pass filtered (40 Hz corner frequency, fourth order, zero phase) the normalized records prior to applying the template matching algorithm. We used templates extracted from each hydrophone for a low‐noise bubble signal (Figure 3), and then used a cross‐correlation matched filter detector (Gibbons & Ringdal, 2006) to detect individual bubble events, with a mean cross‐correlation threshold for all hydrophones of ≥0.8, yielding a catalog with 2,387 bubbles (Figure 2). To avoid detecting reflected signals we set the minimum time between detections at 1.1 s. We experimented with using a variety of different templates (up to 8 templates total), but there was no difference in the timing or number of bubbles detected.

The frequency content of the bubble signals extends from ∼150 Hz up to the 450 Hz resolution limit of our data. The resonant frequency of a bubble can be used to constrain its size (Minnaert, 1933), but it is difficult to identify these resonances in our data because the resonant modes of the borehole, itself, overprint the spectra at frequencies up to ∼250 Hz (Figure 2). It is clear, however, that the bubble signals contain significant energy in the 150–450 Hz band. Assuming a spherical geometry, the relationship between the bubble size and its resonant frequency is given by the Minnaert relationship (Minnaert, 1933):

(1) $$a=\frac{1}{2\pi f}{\left(\frac{3\frac{c_{p}}{c_{v}}P_{A}}{\rho }\right)}^{1/2}$$

where *a* is the bubble radius, *f* is the resonant frequency, *c*_*p*_ and *c*_*v*_ are specific heats (we used a value of $\frac{c_{p}}{c_{v}}=1.405$, assuming hydrogen gas), *P*_*A*_ is the ambient pressure at the source depth (assumed to be 246.6 kPa, since in all cases the bubble signals were first detected on the topmost hydrophone, at a depth of ∼15 m below borehole water level), and *ρ* is the density of water. Using 150 and 450 Hz as limiting values, this relationship yields bubble sizes of 1.1–3.4 cm, which in turn yields total gas mass fluxes through the borehole during our study of 0.078 kg (3.3 × 10^−9^ kg/s) to 0.0029 kg (1.2 × 10^−10^ kg/s). These values represents an unknown fraction of the total free gas content within the local formation. The bubbles are injected into the borehole in swarms, which begin at a fast rate that decreases in an approximately exponential relationship in time, until the swarm ends. We explain this exponential relaxation by deriving a simple model of pressure diffusion when degassing occurs from the rock matrix during a single swarm (Supporting Information S1). All the bubble swarms follow the following relationship:

(2) $$N\left(t\right)=N_{0}\left(1-e^{\alpha t}\right),$$

where *N*_0_ is the number of bubbles in a swarm that occurs over time *t*, and 1/*α* is a relaxation time, in the range 3–8 min (see Supporting Information S1).

## 3 Discussion and Conclusions

A variety of petrological, hydrological, geochemical, and microbiological investigations have been carried out for borehole BA1B (e.g., Cocomazzi et al., 2020; Ellison et al., 2021; Kelemen et al., 2021; Ternieten et al., 2021), providing important context for interpreting our results. We cannot determine the composition of the bubbles we detected on the basis of our acoustic data, but peridotite alteration (Nothaft et al., 2021), and microbial processes supported by alteration products (Schrenk et al., 2013), can both generate H_2_ and CH_4_ gas. These gases have been measured in Hole BA1B fluids, but at all depth intervals the dissolved concentrations were <10% of saturation values (Kelemen et al., 2021). This observation indicates that the bubbles were not exsolved from the borehole fluids, since dissolved concentrations well in excess of saturation values are required to nucleate non‐condensable gas bubbles in a liquid (Jones et al., 1999). Instead, gas bubbles must have migrated laterally into the borehole from the formation, and the injection point into the borehole must have been in the upper ∼30 m since all bubbles were first detected on the top hydrophone. Core analyses have shown that Hole BA1B is crosscut by dense fracture sets at these depths ((Kelemen et al., 2021), Figure 1), and these provide likely pathways for gas migration into the borehole.

Vein filling alteration products in the upper part of the formation include both carbonate and serpentinite minerals. Low‐temperature alteration of previously altered harzburgite in this near‐surface environment has been identified as a means to produce H_2_ gas through the replacement of Fe(II)‐rich brucite with Fe(III)‐bearing serpentine (Kelemen et al., 2021; Ternieten et al., 2021). Additionally, in some of the MBO boreholes, the partial pressure of oxygen (oxygen fugacity, *f*O_2_) recorded by both mineral assemblages and borehole waters at depths of 200–400 m below the surface is on the order of 10^−80^ bars (Kelemen et al., 2021), which is at the limit where H_2_O breaks down to form H_2_. These observations indicate that H_2_ gas generation via ongoing reactions in the peridotite‐hosted aquifer, possibly along with microbial activity, are plausible mechanisms for generating free gas at the study site.

**Figure 4:**
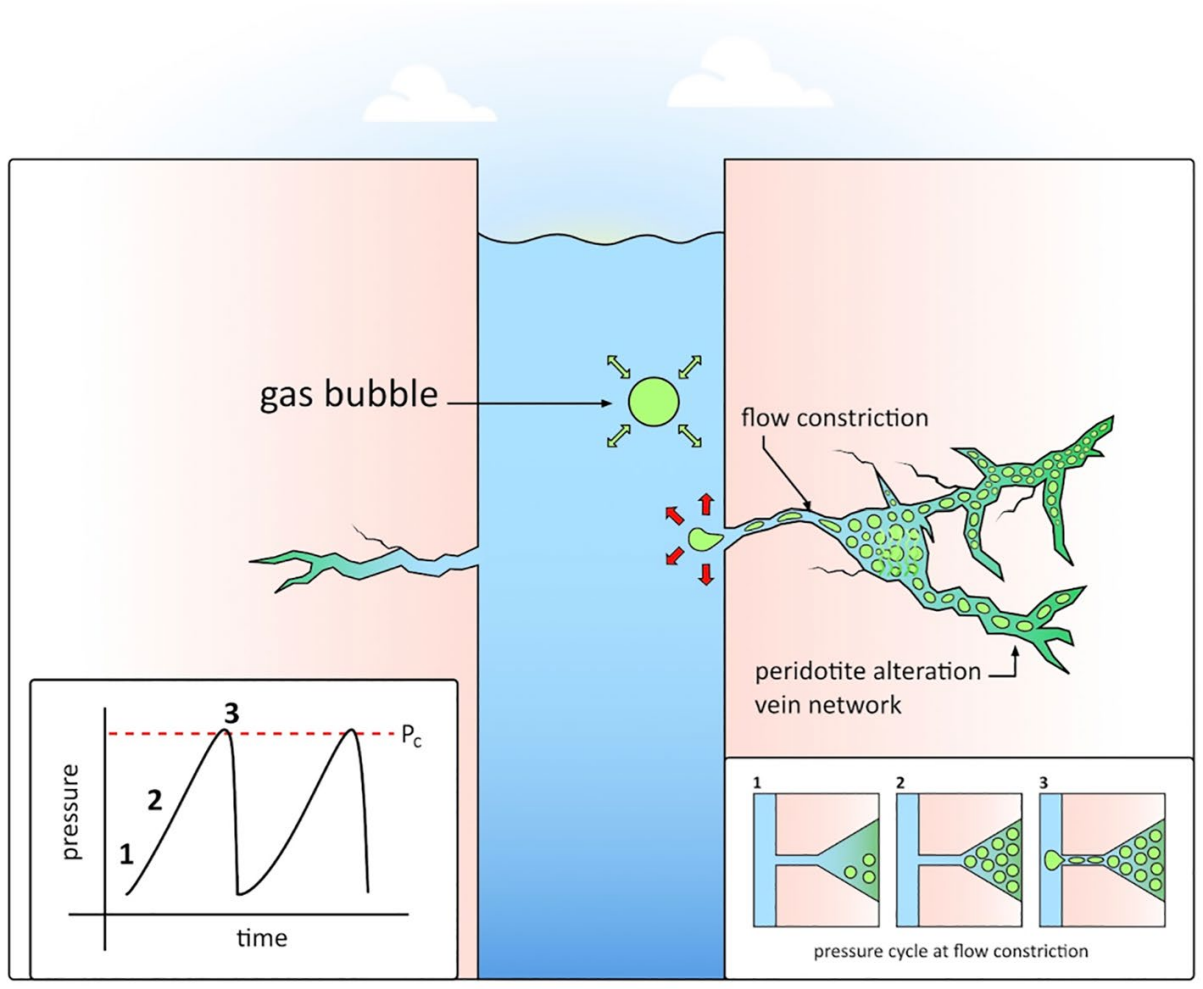
Interpretive schematic. The borehole is intersected by fractures that connect to actively altering rock. The gas generated from alteration accumulates in the fracture network and is pressurized when it reaches a constriction with a high capillary entry pressure. When the gas pressure exceeds a critical value for entering the constriction, *P*_*c*_, it passes through, establishes a connection to the borehole, and the trapped gas decompresses and discharges into the borehole, initially at a fast rate that decreases approximately exponentially until the swarm ends. Water fills the pore spaces previously occupied by gas, and the cycle starts again. Pressurized gas bubbles entering the borehole initially expand, and then oscillate about an equilibrium size, but the acoustic signals detected by the hydrophones (Figure 3) are modified by the dispersive nature of tube wave propagation.

The bubble swarms observed in our acoustic data require that some fraction of the gas generated during peridotite alteration accumulates in the formation as free gas, pressurizes, and migrates rapidly in discrete decompression episodes. These discrete decompression episodes show a relaxation in bubble release similar to experimental two‐phase fluid migrations ((Persoff & Pruess, 1995), see Supporting Information S1). This cyclical degassing will likely prevent the system from reaching a chemical and physical steady state as pressure increases and gas is moved away from gas producing regions. The bubble swarms were not associated with any detectable seismicity, indicating that the decompression mechanism was aseismic and not related to a dynamic fracturing event. Gas migration pathways in bedrock formations are controlled by the fracture network geometry (Heisig & Scott, 2013; Moortgat et al., 2018), which can be complex, and can include constrictions with high capillary entry pressures. Gas can pressurize behind a capillary barrier, and then rapidly decompress when the entry pressure threshold is exceeded and a connection to the atmosphere through the borehole is established (Mumford et al., 2009; Van De Ven & Mumford, 2020), allowing the trapped gas to escape (Figure 4). This is likely similar to the generalized case of gas bubbles growing within any porous media where the total critical gas that allows for free flow is controled by the bubble nucleation fraction and the geometric and topological features of the porous medium (Li & Yortsos, 1995). This process provides the most plausible explanation for our observations, and it represents a previously unknown type of fluid flow during peridotite alteration in natural environments. None of the theoretical approaches (Kelemen & Hirth, 2012), laboratory experiments (e.g., Xing et al., 2018; Zhu et al., 2016), or numerical simulations (e.g., Malvoisin et al., 2021; O. Evans et al., 2020) that have been used to understand peridotite alteration have considered gas accumulation and compression in the flow network. Two‐phase (gas + liquid) fluids have very different flow characteristics compared to single phase (liquid) fluids (Brennen, 2005), and gas could also have a significant impact on the poroelastic behavior and seismic velocity structure of the rock formation (Toksöz et al., 1976; Wang, 2001). Our results highlight that episodic degassing events can keep the gas‐fluid system from maintaining a steady state by removing the gas reaction product produced during the shallow alteration of the peridotite body. More generally, the cycles of gas accumulation, pressurization, and rapid migration required to explain our observations need to be considered in models simulating the chemistry, kinetics, and hydraulic behavior of peridotite alteration in fracture networks.

## Supporting information

Supporting Information S1

## Data Availability

All of the data for the hydrophones have been archived at the Incorporated Research Institutions for Seismology Data Management Center (network code 7F 2019–2020, https://doi.org/10.7914/SN/7F_2019). Please see supplemental python and matlab codes in the associated github repository: https://github.com/mnky9800n/BubblePaper, or on zenodo (https://doi.org/10.5281/zenodo.6832244) https://zenodo.org/badge/latestdoi/470103537.
