# Supplementary material for: Gas Migration Episodes Observed During Peridotite Alteration in the Samail Ophiolite, Oman
Source: Geophys Res Lett. 2022 Oct 31;49(21):e2022GL100395. doi: 10.1029/2022GL100395 (PMC9787822; doi:10.1029/2022GL100395)
Supplement: Supplementary file 1 — Supporting Information S1 [file GRL-49-e2022GL100395-s001.pdf]

Supporting Information for ”Gas migration episodes observed during peridotite alteration in the Samail ophiolite, Oman”

John M. Aiken[1], Robert A. Sohn[2], François Renard[1,3], Juerg Matter[4], Peter Kelemen[5], Bjørn Jamtveit[1]

[1]Njord Centre, Departments of Physics and Geosciences, University of Oslo, PO BOX 1048, Blindern, Oslo, 0316, Oslo, Norway

[2]Department of Geology and Geophysics, Woods Hole Oceanographic Institution, 266 Woods Hole Rd., Woods Hole, 02543, MA, USA

[3]ISTerre, Univ. Grenoble Alpes, Grenoble INP, Univ. Savoie Mont Blanc, CNRS, IRD, Univ. Gustave Eiffel, 38000, Grenoble, France

[4]School of Ocean and Earth Science, University of Southampton, Southampton,Southampton, UK

[5]Lamont Doherty Earth Observatory, Columbia University, 61 Rte 9W, Palisades, 10964, New York, USA

Contents of this file

1. Text S1
2. Figures S1 to S3
3. Tables S1

Introduction

In this supplemental, we describe the inter-event timing of bubbles during each bubble swarm. We derive an exponential law for the inter-event timings. We then fit this law to the degassing swarm events. This exponential law provides theoretical evidence for the relaxation observed during each degassing event, which starts with a fast release of bubbles and then a slower relaxation until no more bubbles are released.

Text S1.

Here, we develop a simple one-dimensional model of gas diffusion into the peridotite matrix that reproduces the exponential form of the inter-event time of bubble release in each swarm. We start with mass conservation:

$$\frac{\delta \rho}{\delta t} + \nabla(\rho \bar{v}) = 0, \tag{1}$$

where ρ is the material density, t is time, and $\bar{v}$ is the mean gas velocity (or the velocity field), assuming that the process is isothermal (constant temperature), i.e. the ideal gas law applies (as stated in the paper):

$$P = \rho R T, \tag{2}$$

where P is pressure, ρ is the material density, R is a constant, and T is temperature. Therefore, we can relate the pressure to the velocity via:

$$\frac{\delta P}{\delta t} + \nabla(P \bar{v}) = 0. \tag{3}$$

If we assume the flow follows Darcy's law:

$$\bar{v} = -k\nabla P, \tag{4}$$

where k is the permeability of the host rock, we can combine Eqn. 3 and Eqn. 4 to obtain:

$$\frac{\delta P}{\delta t} - \nabla(kP\nabla P) = 0. \tag{5}$$

If we consider only small variations in P, we write $P = P_0 + \epsilon P_1$ and we get:

$$\frac{\delta P_1}{\delta t} - kP_0\nabla^2 P_1 = 0, \tag{6}$$

where we can write $D = kP_0$. We can now use this relationship to represent the degassing of a simplified one-dimensional porous reservoir, as represented in Fig. S1.

We can now define the change in pressure over time and space, that is, the pressure required to degas through the permeable area from the gas reservoir at a distance X from the borehole, as:

$$\frac{\delta P_1}{\delta t} = D\nabla^2 P_1 = D\frac{\delta^2 P_1}{\delta x^2}. \tag{7}$$

Solving this equation by separation of variables, we get:

$$P_1 = T(t)X''(x)\frac{1}{D}\frac{T'}{T} = \frac{X''}{X}, \tag{8}$$

which gives with separation constant λ:

$$\frac{X''}{X} = -\lambda_n, \tag{9}$$

and thus we can define X as:

$$X(x) = \sum_{n=0}^{\infty} A_n \sin(\lambda_n x), \tag{10}$$

where

$$\lambda_n = \frac{\pi(n+\frac{1}{2})}{h}$$
$$P_1(x_1, t=0) = X(x) = P_i$$
$$A_n = \int_0^h P_i \sin(\lambda_n x) dx$$
$$= \frac{P_i h}{\pi(n+\frac{1}{2})}$$
$$= \frac{P_i}{\lambda_n}.$$

The time is:

$$T(t) = e^{-\lambda_n D t} \tag{11}$$

Thus, the overall solution for the pressure required to flow gas at a distance x over the system for time t becomes:

$$P_1(x,t) = \sum_{n=0}^{\infty} \frac{P_i}{\lambda_n} e^{-\lambda_n D t} \sin(\lambda_n x) \tag{12}$$

where:

$$\lambda_n = \frac{\pi(n+\frac{1}{2})}{h}. \tag{13}$$

Thus, the gas pressure evolution follows an exponential relaxation, which is seen in Fig. S2.

Using these relationships, we can also derive the total mass produced during a degassing event:

$$M(t) = \int_0^h \rho(x,t)dx = \int_0^h \frac{P(x,t)}{RT}dx \tag{14}$$

and

$$\begin{aligned} &= \frac{1}{RT}\int_0^h (P_0 + P_1)dx \\ &= \frac{P_0 h}{RT} + \frac{1}{RT}\int_0^h P_1(x,t)dx. \end{aligned}$$

Taking the derivative with respect to time, we get:

$$\frac{dM}{dt} = \frac{P_i}{RT}(-D\sum_{n=0}^{\infty})e^{-\lambda_n DT}\int_0^h \sin(\lambda_n x)dx \tag{15}$$

$$\begin{aligned} &= \frac{P_i}{RT}(\frac{-D}{\lambda_0}e^{-\lambda_0 Dt}) \\ &= -\frac{D\rho_i}{\lambda_o}e^{-\lambda_0 Dt}. \end{aligned}$$

Thus, the total mass released during a degassing event follows:

$$M_{release} = M_0(1 - e^{-\lambda_0 Dt}). \tag{16}$$

Given that the bubbles are more or less equal in size, as calculated by their Minnaert radius, we can rewrite Eqn. 16 to be a count density instead:

$$N(t) = N_0(1 - e^{\alpha t}), \tag{17}$$

where

$$\alpha = \lambda_0 D = \lambda_0 k P_0, \qquad (18)$$

and α is used as the fitting parameter to calculate the relaxation time represented in Fig. S2. For most bubble swarms (See Table S1), the parameter α varies in the range 0.002-0.006 s^{-1}, which corresponds to relaxation times, $1/\alpha$, in the range 3-8 minutes. Using this relationship across all degassing events we can see the red curve for the median α value (taken from fits to all degassing events) in Fig. S3.

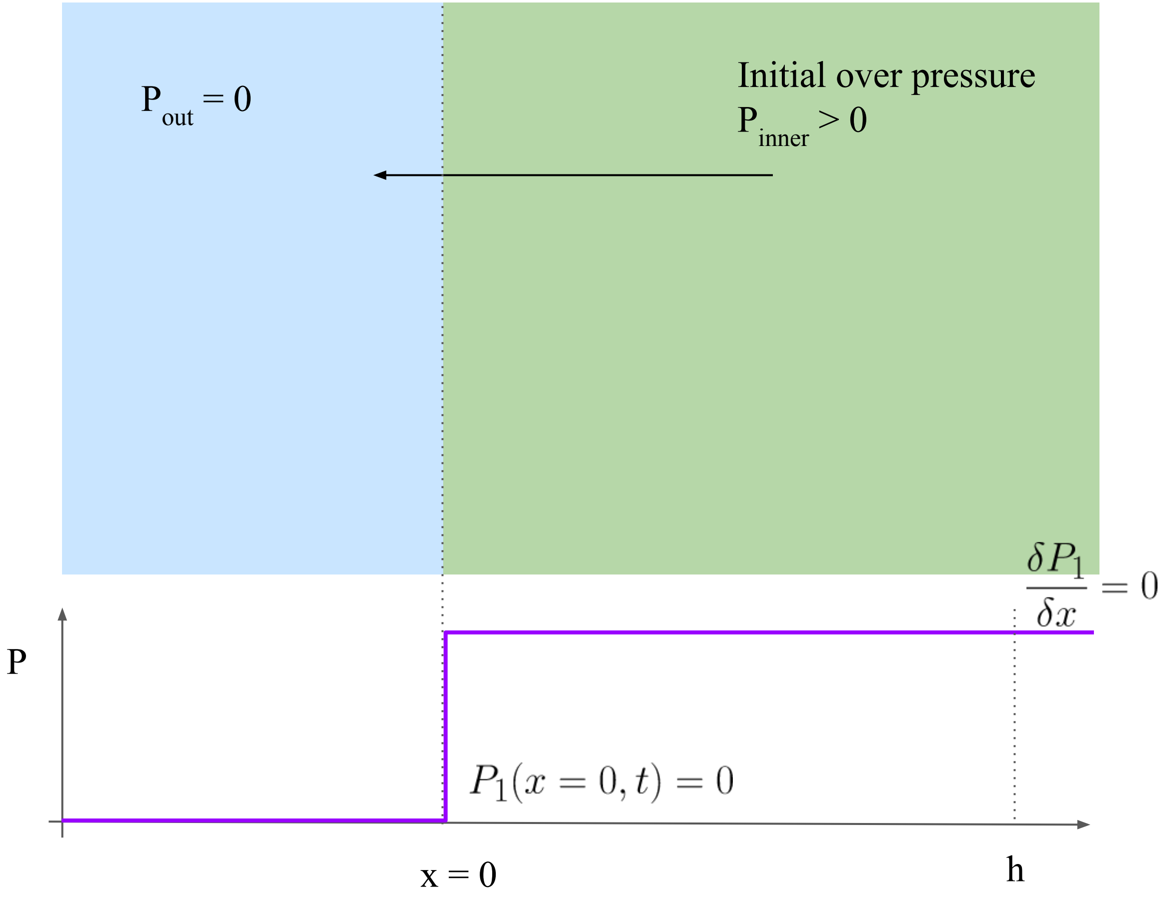

Figure S1. Conceptual diagram of one-dimensional gas flow.

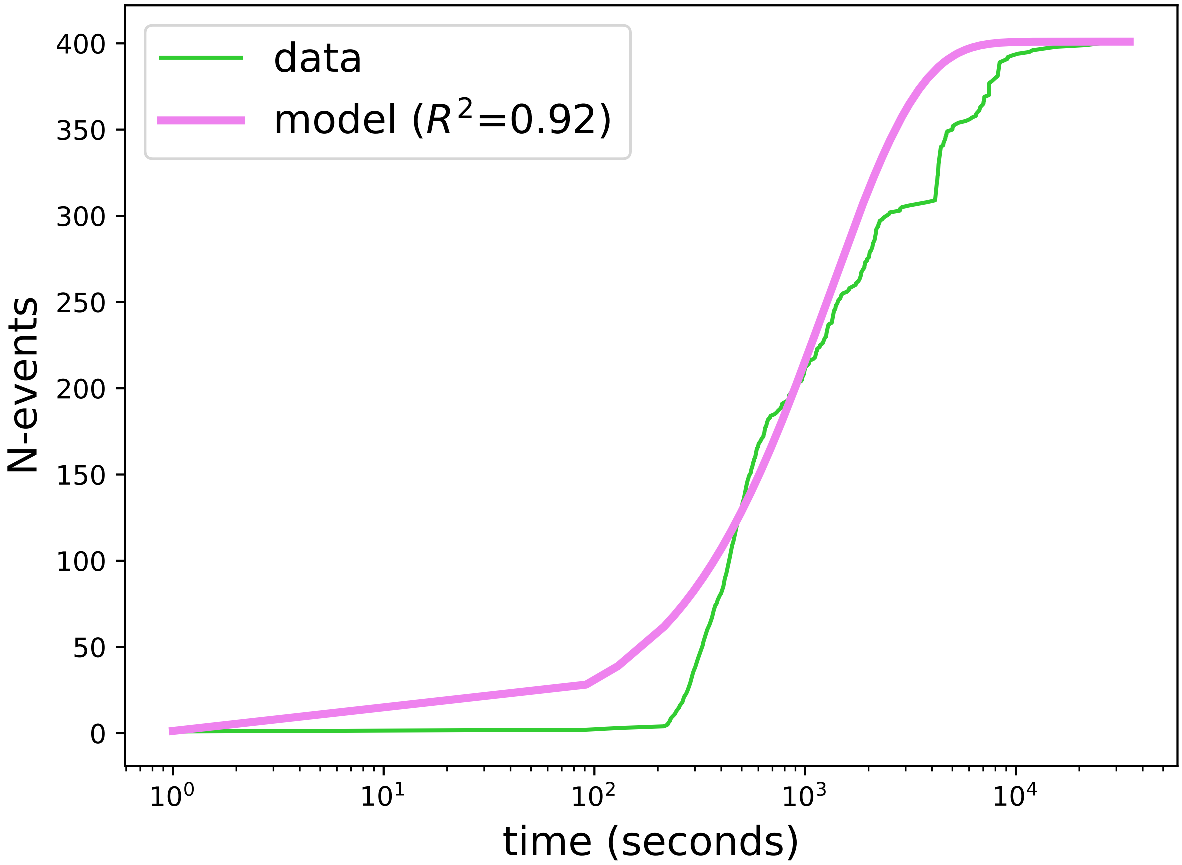

Figure S2. Example of relaxation for a single degassing event (green curve) on May 18, 2019, which corresponds to the first initial swarm shown in Fig. 2. The pink curve shows a fit using an exponential relaxation derived in Eq. 17, with model parameters α = 8.133e-4, and N_0 = 380.

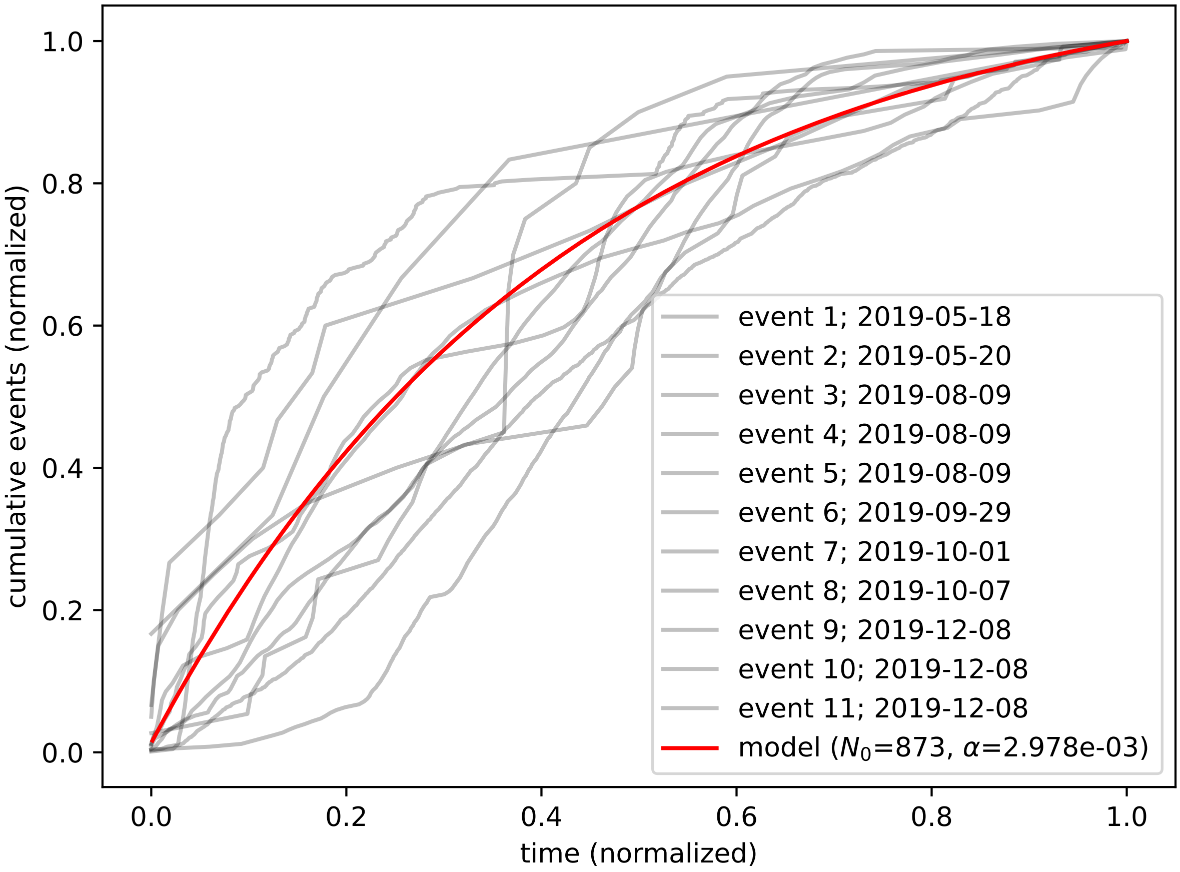

Figure S3. Cumulative event counts of degassing events per time unit. Both values are normalized to 1. The red line indicates the model using the median fit values for the count density calculated using Eqn. 17.

**event number**	**event selection window**	**event count**	α	R^2
1	2019-05-18 11:30:00 - 14:00:00	380	8.133e-4	0.935
2	2019-05-20 13:35:30 - 14:20:00	871	9.500e-4	0.845
3	2019-08-09 11:00:00 - 12:15:00	82	3.500e-3	0.985
4	2019-08-09 13:00:00 - 13:15:00	103	6.500e-3	0.875
5	2019-08-09 13:15:00 - 14:00:00	243	2.500e-3	0.602
6	2019-09-29 11:00:00 - 11:30:00	37	5.000e-4	0.859
7	2019-10-01 12:00:00 - 13:00:00	214	4.000e-3	0.627
8	2019-10-07 10:55:00 - 11:05:00	15	1.419e-2	0.906
9	2019-12-08 00:00:00 - 01:00:00	20	2.000e-3	0.804
10	2019-12-08 12:50:00 - 13:00:00	6	1.500e-2	0.868
11	2019-12-08 13:30:00 - 15:30:00	87	3.907e-4	0.969

Table S1. Time windows for degassing events in the hydrophone data. Event counts represent the number of bubbles in each event. These do not add up to 2387 bubbles because there are some bubbles that occur during the periods of general quiescence. α-values represent the power law fit parameter α in Eqn. 17. R^2 also is shown to represent the goodness of fit of the power law to the data.
